# Environmental Exposure to Micro- and Nanoplastics: Linking Cardiovascular Disease and Cancer Through Shared Biological Pathways—A Critical Review

**DOI:** 10.3390/antiox15070786

**Published:** 2026-06-24

**Authors:** Andrea Borghini, Mariangela Palazzo, Alessandro Tonacci, Fabrizio Minichilli, Haotian Wu, Francesca Gorini

**Affiliations:** 1 Institute of Clinical Physiology, National Research Council, 56126 Pisa, Italy; mariangelapalazzo@cnr.it (M.P.); alessandro.tonacci@cnr.it (A.T.); fabrizio.minichilli@cnr.it (F.M.); 2 Department of Environmental Health Sciences, Columbia University Mailman School of Public Health, New York, NY 10032, USA; hw2694@cumc.columbia.edu

**Keywords:** microplastics, nanoplastics, cardiovascular disease, cancer, oxidative stress, inflammation, immune dysregulation, genetics, gut microbiota

## Abstract

Micro- and nanoplastics (MPs/NPs) are ubiquitous environmental contaminants increasingly detected in air, food, drinking water, and human tissues, raising concerns about their potential long-term health effects. Accumulating evidence indicates that these particles can enter the human body, cross biological barriers, and elicit cellular and molecular responses relevant to disease development. This review synthesizes current mechanistic evidence linking MP/NP exposure to cardiovascular disease (CVD) and cancer, two leading global causes of morbidity and mortality that share interconnected pathogenic pathways. Key mechanisms include chronic inflammation, oxidative stress, gut microbiota dysbiosis, genotoxicity, and epigenetic alterations, all of which are widely implicated in both conditions. However, the available evidence is still largely derived from in vitro and animal studies, with limited human epidemiological data. Important uncertainties remain regarding real-world exposure characterization, dose–response relationships, and long-term clinical outcomes, underscoring the need for standardized analytical approaches, validated exposure and effect biomarkers, and large-scale longitudinal studies to clarify causal associations for both cancer and CVD. Taken together, current evidence suggests that MPs/NPs may represent emerging environmental contributors to shared pathogenic pathways linking CVD and cancer; however, establishing causality in humans will require well-designed longitudinal studies that integrate exposure assessment and clinical outcomes.

## 1. Introduction

Global plastic production has increased exponentially over recent decades, rising from 234 million tons (Mt) in 2000 to 460 Mt in 2019, with no indication of a future decline [1,2]. Owing to their favorable physicochemical properties—including versatility, durability, hydrophobicity, low density, and low production costs—plastics are used across nearly all industrial and consumer sectors, with demand further amplified during the COVID-19 pandemic [3,4]. Consequently, plastics have become ubiquitous in all environmental compartments, and their waste now represents a persistent and pervasive pollutant [5]. Global plastic use is projected to reach 884 Mt by 2050, with waste generation expected to double over the same period, even if packaging recycling rates exceed 75% [1].

Microplastics (MPs) and nanoplastics (NPs), generated through the degradation of plastic waste, have emerged as major environmental and public health concerns due to their widespread distribution across ecosystems and their potential to infiltrate human tissues and barriers [5,6]. Although MPs and NPs are often considered relatively inert materials, they can absorb substances such as additives, heavy metals, proteins, and microorganisms onto their surfaces, potentially increasing their toxicity [7]. While the environmental impact of MPs and NPs is well documented, their effects on human health remain incompletely understood due to uncertainties related to exposure routes, detection methods, and toxicity evaluation [6,8]. However, recent evidence suggests that plastic particles are increasingly detected in various human organs and may trigger adverse biological responses, including inflammation, oxidative stress, and cytotoxicity, with possible serious health consequences [8,9]. Such biological alterations are recognized contributors to the development of chronic non-communicable diseases, including cancer and cardiovascular disease (CVD), two of the leading causes of global morbidity and mortality [10]. Shared mechanisms include chronic inflammation, oxidative stress, immune dysregulation, clonal hematopoiesis, gut dysbiosis, and genetic and epigenetic alterations [10,11].

Given the growing body of evidence—largely derived from preclinical studies—showing that plastic particles may adversely affect the cardiovascular system and influence biological pathways involved in tumor initiation and progression, this review aims to provide an integrated overview of the current mechanistic evidence. In particular, it explores how MPs and NPs may trigger or exacerbate biological pathways that are commonly implicated in both CVD and cancer.

To this end, a comprehensive literature search was conducted in the PubMed database to identify studies published in English between January 2000 and April 2026 that investigated the potential role of MPs and NPs in CVD and cancer. The search strategy combined terms related to plastic particles (“microplastics”, “nanoplastics”) with terms associated with cardiovascular and oncological outcomes (“cardiovascular disease”, “atherosclerosis”, “thrombosis”, “cancer”, “carcinogenesis”) and shared pathogenic mechanisms (“inflammation”, “oxidative stress”, “gut dysbiosis”, “genotoxicity”, “epigenetic modifications”, and “cellular senescence”). In addition, the reference lists of relevant articles were screened to identify further eligible studies.

Beyond summarizing the mechanisms underlying MP/NP-associated cardiovascular and cancer risk, with particular attention to the role of antioxidant defenses in modulating plastic-induced adverse effects, this review also seeks to highlight current research gaps and propose future directions for risk assessment and translational investigations.

## 2. Microplastics and Nanoplastics: Sources, Characteristics, and Routes of Human Exposure

MPs are commonly defined as plastic particles with a diameter smaller than 5 mm and originate from the progressive degradation of larger plastic materials [12,13]. This process can occur over hundreds or even thousands of years, depending on the physicochemical properties of the plastics and the surrounding environmental conditions, and involves a complex interplay of mechanisms including ultraviolet radiation, mechanical abrasion, temperature fluctuations, and, to a lesser extent, biodegradation [7,14]. Besides resulting from the degradation of larger plastic waste (secondary MPs), primary MPs can also be deliberately produced for consumer and commercial uses, such as cleaning products, cosmetics, drug delivery systems, and sandblasting applications [7]. Polyethylene (PE), polypropylene (PP), and polystyrene (PS) are among the most prevalent polymers detected in environmental microplastic contamination [7]. The small size of MPs, which confers a high surface-to-volume ratio and enhanced mobility, enables them to act as “Trojan horses,” facilitating the transport of heavy metals, persistent organic pollutants, plastic additives (e.g., phthalates and bisphenol A—BPA), harmful pathogens, and engineered nanoparticles [7,15,16]. Furthermore, plastic particles exhibit a wide range of morphologies, and differences in shape (e.g., fibers, foams, cylindrical or spherical beads, and granules) critically modulate their interactions with biological surfaces, leading to heterogeneous cellular and tissue responses [17].

Notably, despite being widely distributed in the environment, MPs are subject to bioaccumulation and potentially biomagnification along aquatic food webs [18]. Accordingly, human exposure to MPs occurs primarily through the consumption of seafood, marine salt, vegetables, honey, and mineral water, as well as through ingestion from plastic food packaging [5,18]. In addition, exposure occurs through the inhalation of outdoor air, particularly in urban environments, and indoor dust containing MPs, while dermal contact via cosmetics and personal care products appears to represent a comparatively minor exposure route [4,5,6]. Annual human intake of MPs from food is estimated to reach approximately 1.42 × 10^5^–1.54 × 10^5^ particles per capita, an amount considered equivalent to the ingestion of about 50 plastic bags [19]. Plastic microparticles can further fragment into NPs, typically defined as particles smaller than 1 µm [7]. However, unlike their microplastic counterparts, current research on NPs has mainly focused on aquatic systems, and knowledge of their potential impacts on human health—although exposure may occur through the food chain—remains limited [7,14].

**Figure 1:**
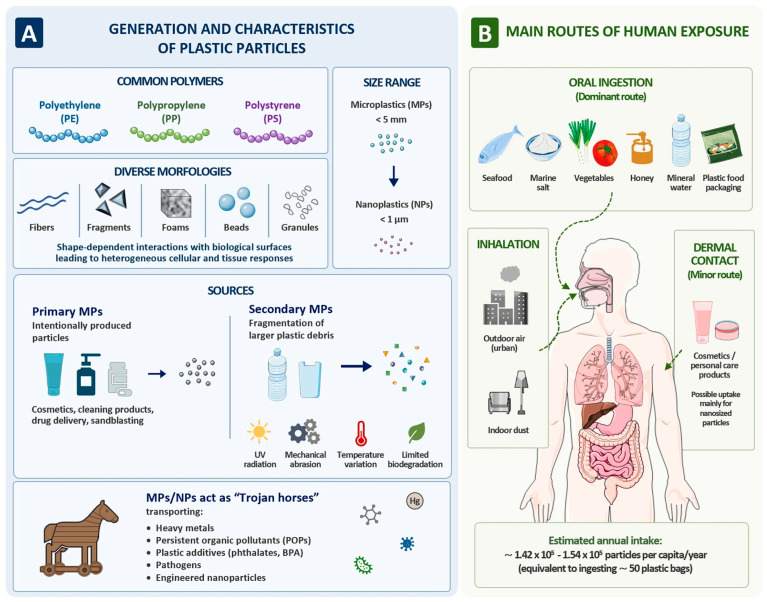
Generation, characteristics, and human exposure pathways of microplastics and nanoplastics. (**A**) Overview of the origin and physicochemical characteristics of plastic particles. Common polymers include polyethylene (PE), polypropylene (PP), and polystyrene (PS). Plastic debris occurs in different morphologies (fibers, fragments, foams, beads, and granules) and spans a broad size range, from microplastics (MPs; <5 mm) to nanoplastics (NPs; <1 μm). MPs may originate as primary particles intentionally manufactured for industrial or commercial applications or as secondary particles generated through the fragmentation of larger plastic items by environmental weathering processes, including UV radiation, mechanical abrasion, temperature fluctuations, and limited biodegradation. Owing to their large surface area and physicochemical properties, MPs and NPs can also act as “Trojan horses,” adsorbing and transporting co-contaminants such as heavy metals, persistent organic pollutants (POPs), plastic additives, pathogens, and engineered nanoparticles. (**B**) Main routes of human exposure to MPs and NPs. Oral ingestion represents the predominant exposure pathway through contaminated food and beverages, including seafood, marine salt, vegetables, honey, and drinking water, as well as through migration from plastic food packaging. Additional exposure occurs through inhalation of airborne particles from outdoor and indoor environments, whereas dermal contact is considered a minor route, mainly relevant for nanosized particles present in cosmetics and personal care products. Once internalized, plastic particles may interact with multiple organ systems and contribute to biological responses associated with human health effects. Image adapted from Servier Medical Art (https://smart.servier.com (accessed on 10 May 2026)), licensed under CC BY 4.0 (https://creativecommons.org/licenses/by/4.0/ (accessed on 10 May 2026)).

Once ingested, the absorption and translocation of MPs/NPs across the gastrointestinal tract largely depend on particle size [16]. Particles smaller than 150 µm have the potential to penetrate the intestinal epithelium and enter the systemic circulation, whereas most particles larger than 150 µm are eliminated via feces [16]. A small fraction of these larger particles may be retained in the gut, where they can potentially alter the composition of the colonic microbial community [16,20]. Similarly, inhalation represents an additional exposure route, with the entry of MPs/NPs into the respiratory system being largely restricted to the lower airways for particles with aerodynamic diameters smaller than 5 µm [21]. Notably, particles in the submicron range can penetrate the alveolar region, traverse the pulmonary epithelial barrier, and subsequently enter the systemic circulation [16]. In parallel, although the ability of plastic particles to cross the skin barrier remains a matter of debate, the direct application of cosmetics containing MPs/NPs may allow nanosized particles to be absorbed via percutaneous uptake and, upon dermal entry, reach the dermis and gain access to the bloodstream or lymphatic system, thereby potentially enabling systemic distribution [22,23] (Figure 1).

**Figure 2:**
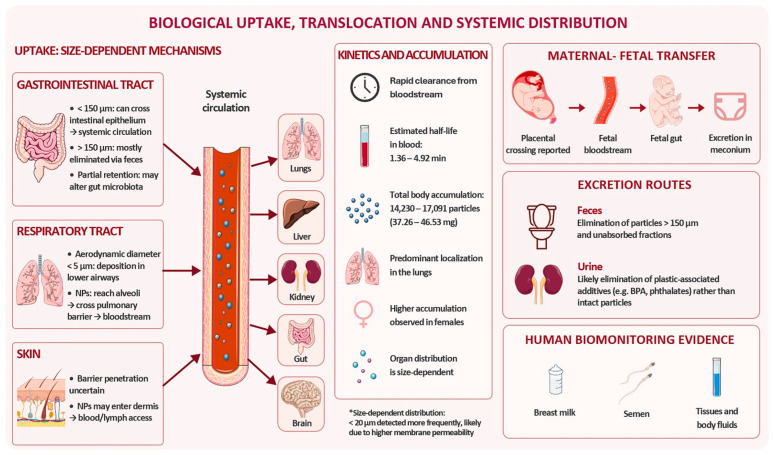
Biodistribution and potential elimination pathways of microplastics and nanoplastics in the human body. This schematic representation illustrates the potential systemic distribution of MPs/NPs following human exposure, including their translocation across biological barriers and possible presence in different organs. Plastic particles have been reported in multiple tissues and body fluids, reflecting exposure and biodistribution processes; however, such findings should not be interpreted as evidence of fully established or generalized pathways of translocation and accumulation. Potential routes of elimination include fecal excretion and urinary clearance, mainly related to associated compounds rather than intact particles. Current evidence on biodistribution and elimination is heterogeneous and largely derived from experimental models and limited human studies and may vary depending on particle size, physicochemical properties, and analytical detection methods. Therefore, the depicted processes should be interpreted as indicative of possible distribution patterns and kinetic behavior rather than definitive evidence of biological effects or causal relationships with disease. Image adapted from Servier Medical Art (https://smart.servier.com (accessed on 10 May 2026)), licensed under CC BY 4.0 (https://creativecommons.org/licenses/by/4.0/ (accessed on 10 May 2026)).

Of note, beyond being detected in a variety of human tissues and body fluids, including breast milk and semen, MPs may also cross the placental barrier, enter the fetal bloodstream, and ultimately reach the fetal gut, where they can be excreted in meconium [17,24]. In addition, their detection in the olfactory bulb suggests the existence of a potential translocation pathway to the brain [17,25]. MPs and NPs exhibit size-dependent clearance and biodistribution, with overall in vivo elimination occurring on the order of hours, as evidenced by experimental tracking studies in animal models [26]. Recent estimates indicate that total MP accumulation may range from 14,230 to 17,091 particles, corresponding to a mass of 37.26–46.53 mg, with predominant localization in the lungs and higher accumulation observed in females compared with males [27]. Organ-specific distribution appears to be size-dependent, with particles smaller than 20 µm detected in greater abundance, likely reflecting differences in the permeability of biological membranes [28,29]. Furthermore, in addition to fecal elimination, MPs/NPs or their associated additives may also be excreted via urine, a process hypothesized to primarily reflect the renal clearance of plastic-associated contaminants, such as BPA and phthalates, commonly used in food packaging and personal care products, rather than intact particles [8] (Figure 2).

Overall, the ubiquitous environmental presence of MPs/NPs, combined with multiple exposure routes and evidence of systemic distribution and organ accumulation, highlights their potential to interfere with biological processes relevant to human health. However, despite increasing evidence of widespread exposure and systemic distribution, current estimates of human MP/NP exposure remain highly uncertain due to methodological heterogeneity and the lack of standardized analytical approaches.

Therefore, to ensure scientific rigor in the interpretation of the available evidence, it is important to distinguish between different levels of evidence. In this review, we therefore differentiate between: (i) detection of particles in human tissues, which reflects exposure; (ii) associations with clinical or biological markers, which are observational and do not imply causality; (iii) mechanistic evidence derived from in vitro and in vivo experimental models; and (iv) evidence of causality in humans, which is currently lacking. Based on this framework, the following sections will focus on mechanistic insights derived from experimental models, while human evidence is discussed in terms of exposure and association rather than causation.

## 3. Potential Shared Mechanisms Linking Microplastic/Nanoplastic Exposure, Cancer and Cardiovascular Disease

CVD and cancer remain the leading causes of morbidity and mortality worldwide [30]. According to the World Health Organization, CVD accounted for approximately 19.8 million deaths in 2022, representing about 32% of all deaths [31]. In the same year, the International Agency for Research on Cancer estimated around 20 million new cancer cases and 9.7 million cancer-related deaths [32]. Although long viewed as separate contributors to mortality, growing evidence indicates that CVD and cancer are closely interconnected and frequently coexist within a complex and bidirectional relationship in which each condition can distinctly influence the development, progression, and outcomes of the other, thereby significantly affecting long-term survival [30]. Thus, despite being distinct clinical entities, CVD and cancer tend to overlap, with this interplay driven by shared risk factors and multiple convergent pathophysiological processes [10]. Chronic systemic inflammation, oxidative stress, metabolic dysregulation, intestinal dysbiosis, epigenetic modifications and genetic instability, cellular senescence, and clonal hematopoiesis have been identified as shared mechanisms underlying both CVD and cancer [10,11,33].

As discussed in the previous section, once MPs/NPs enter the human body, they can disseminate systemically via the circulatory system to multiple organs and, at relatively low concentrations in experimental settings, may induce oxidative stress, leading to the production of pro-inflammatory cytokines, apoptosis, cytotoxicity, and alterations in gene expression [34].

An increasing body of evidence indicates that plastic particles are present and accumulate in human cardiovascular tissues as well as in tumor tissues. MPs have been identified in cardiac and pericardiac tissues of patients undergoing cardiac surgery, predominantly composed of polyethylene terephthalate (PET) and polyurethane (PU), which together account for approximately 90% of the total MPs detected, with particle sizes ranging from 20 to 469 μm [35]. However, differences in MP size and composition observed between pre- and post-surgical blood samples suggest that the use of medical devices containing plastic components during surgery may have influenced the detected MP distribution [35]. In line with these findings, MPs—including polyamide 66 (PA66), polyvinyl chloride (PVC), and PE—have been detected in 80% of thrombus samples from patients undergoing arterial or venous thrombectomy for ischemic stroke, myocardial infarction (MI), or deep vein thrombosis [36]. Median MP concentrations were 61.75 μg/g, 141.80 μg/g, and 69.62 μg/g, respectively, and a positive association was observed between disease severity and MP concentration within thrombi [36]. Furthermore, MPs (mean concentration: 118.66 μg/g), with PET, PA-66, PVC, and PE being the predominant components, were significantly enriched in atherosclerotic coronary and carotid plaques compared with plaque-free aortic tissue, suggesting an association with atherosclerosis [37]. Consistently, the presence of plastic particles within carotid plaques—mainly PE and PVC, with mean concentrations of 21.7 and 5.2 μg/g per plaque, respectively—has been associated with a 4.5-fold increased incidence of non-fatal MI, stroke, or all-cause mortality [38]. Furthermore, PVC, detected in 95.4% of blood samples from patients undergoing coronary angiography for MI, was significantly associated with a 9% increase in the risk of major adverse cardiac events, following a dose–response relationship [39]. This finding further corroborates the emerging links between MP/NP exposure or accumulation, cardiovascular risk markers, and adverse clinical outcomes. Yang and colleagues reported a correlation between MP exposure and vascular pathology complexity in patients with acute coronary syndrome (ACS) [40]. Blood MP concentrations, with PE present at the highest levels, were significantly higher in ACS patients compared to controls (161.65 µg/g vs. 100.13 µg/g) and were further elevated in patients with MI compared to those with unstable angina [40]. Notably, while low-risk ACS patients showed no significant association with MP exposure, this relationship became significant in medium- to high-risk groups, suggesting a potential prognostic value of MP levels in ACS [40].

Although human studies investigating the relationship between MP/NP exposure and cancer development are still in their infancy, MPs have been detected in several human tumors. Zhao et al. [41] recently identified MPs in 42.6% of tumor samples from patients who had not received prior treatment. PS was the predominant polymer, with a mean concentration of 59.56 ng/g, followed by PVC (51.98 ng/g) and PE (86.94 ng/g) [41]. Lung cancer exhibited the highest detection rate (80%), whereas pancreatic, colorectal, gastric, and cervical tumors showed rates of 70%, 50%, 40%, and 17%, respectively [41]. Notably, MPs were not found in esophageal tumor specimens, suggesting heterogeneous adhesive affinities of MPs across tumor types, potentially reflecting site-specific binding properties or, alternatively, differences in clearance efficiency among tumors [41]. Nine types of MPs—predominantly PE, PP, and PVC—have also been detected in over 85% of penile cancer samples, with an average abundance of 6.42 particles per gram and most particles falling within the 20–50 µm size range [42]. Additionally, MPs not only showed a higher detection rate and greater abundance in cancerous tissue compared with adjacent normal tissue but were also characterized by a richer and more diverse polymeric profile [42]. Consistently, the mean abundance of MPs in human prostate samples was 290.3 µg/g in tumor tissue and 181.0 µg/g in para-tumor tissue, with particle diameters ranging from 20 to 50 µm [43]. Comparable findings have also been reported for colorectal cancer (CRC) (with PE, PET, PP, PS, and PVC as the most prevalent particle types) and cervical cancer (with PE, polyethylene-co-polypropylene, and PP as the predominant types), with significant differences between tumor and adjacent non-tumor tissues [9,44]. Moreover, a recent case–control study offered the first epidemiological indication of a possible association between MP exposure and CRC, reporting significantly higher fecal MP concentrations in CRC patients compared with controls and an approximately 11-fold increase in CRC risk among individuals with higher fecal MP levels [45]. However, the presence of MPs/NPs in cardiovascular or tumor tissues should not be interpreted as evidence of disease causation. Rather, current findings indicate associations with biological processes implicated in disease pathogenesis. While these mechanisms provide biologically plausible links between MP/NP exposure and adverse health outcomes, the extent to which they contribute to the initiation, progression, or severity of CVD and cancer in humans remains to be established.

**Figure 3:**
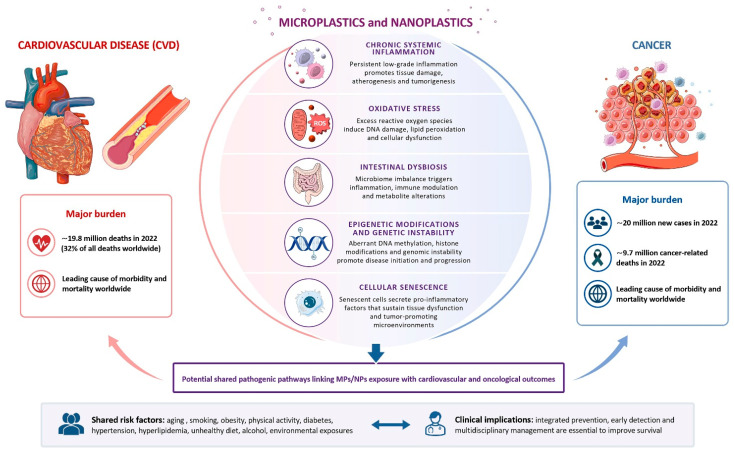
Potential biological pathways associated with MP/NP exposure and relevant to cardiovascular and oncological disease processes. This schematic illustration summarizes biological mechanisms that may be triggered or modulated by MP/NP exposure, including oxidative stress, chronic inflammation, gut dysbiosis, genotoxicity, and epigenetic modifications. These interconnected processes are widely implicated in the pathophysiology of cardiovascular disease and cancer. Current evidence is largely derived from experimental models, while human data are primarily associative; therefore, these pathways should be interpreted as biologically plausible mechanisms rather than evidence of causal relationships. Image adapted from Servier Medical Art (https://smart.servier.com (accessed on 11 May 2026)), licensed under CC BY 4.0 (https://creativecommons.org/licenses/by/4.0/ (accessed on 11 May 2026)).

In the following sections, we examine how MPs/NPs may be associated with the risk of both CVD and cancer by potentially acting through the shared etiopathogenic mechanisms that underpin these two distinct disease groups (Figure 3). Importantly, the interpretation of these mechanisms requires integration with established organ-level physiology. Molecular and cellular alterations triggered by MP/NP exposure may converge on key pathophysiological processes relevant to both CVD and cancer. In the cardiovascular system, these include endothelial dysfunction, impaired nitric oxide (NO) bioavailability, vascular inflammation, thrombosis, and myocardial remodeling. In parallel, in the oncological context, these alterations may influence tumor initiation and progression through effects on cell proliferation, survival signaling, immune evasion, and the tumor microenvironment. This integrative framework is essential for linking experimental findings to clinically relevant disease processes.

Furthermore, the biological effects of MPs/NPs may vary depending on the route of exposure, which determines their toxicokinetics, including absorption, distribution, and interaction with specific tissues and biological systems. Oral, inhalation, and dermal exposures are associated with distinct pathways of particle uptake and organ-specific interactions, which should be considered when interpreting mechanistic findings.

In this review, MPs are defined as particles <5 mm, while NPs are defined as particles <1 μm, acknowledging that the boundary between small MPs and NPs may vary depending on analytical methods and study-specific definitions. “MPs/NPs” is used as a collective term for readability, while specific effects are discussed based on the original experimental conditions. Furthermore, while MPs and NPs are often discussed under a combined framework in the literature, they exhibit substantial differences in size-dependent biological behavior, including cellular uptake, biodistribution, and toxicity. Where possible, evidence has been stratified according to particle type; however, in cases where studies do not clearly distinguish between MPs and NPs, findings have been interpreted within a size-dependent conceptual framework. Additionally, it should be noted that most of the experimental evidence discussed in the following sections derives from controlled laboratory studies using well-defined MPs and NPs, whereas studies using environmentally derived particles are less frequent and may introduce additional confounding factors.

### 3.1. Inflammation

Chronic inflammation plays a central role in the pathogenesis of CVD, as persistent low-grade systemic immune activation drives atherogenesis and increases the risk of hypertension, hyperlipidemia, and type 2 diabetes, well-established CVD risk factors [11,33]. In parallel, sustained activation of pro-inflammatory signaling pathways also promotes cancer initiation and progression by enhancing tumor cell proliferation, survival, and metastatic potential, processes that are further facilitated by inflammation-induced immune suppression [11,33].

Exposure to MPs/NPs has been increasingly associated with persistent inflammatory responses characterized by elevated pro-inflammatory cytokines, oxidative stress, and activation of key inflammatory signaling pathways [46,47]. Recent evidence indicates that MPs/NPs have been shown to modulate the expression of senescence markers such as p16, p21, and senescence-associated β-galactosidase (SA-β-gal) activity, together with the development of a senescence-associated secretory phenotype (SASP) that further amplifies inflammatory signaling [47]. This inflammation–senescence axis has been increasingly associated with the pathogenesis of age-related diseases, including cardiovascular, neurodegenerative, metabolic, and chronic inflammatory disorders, suggesting that MP/NP exposure may be involved in biological processes relevant to disease development [47]. Nevertheless, it is important to distinguish between nonspecific particle-induced immune activation and mechanisms that are causally implicated in disease development. While many studies report increased cytokine production, oxidative stress, and activation of inflammatory pathways, these responses are common to a wide range of environmental exposures and do not necessarily translate into pathological outcomes. Accordingly, the pathological relevance of MP/NP-induced inflammation depends on whether these responses are sustained and functionally linked to processes such as endothelial dysfunction, atherogenesis, thrombosis, vascular remodeling, or tumor-promoting microenvironments.

Evidence linking MP/NP exposure to inflammation derives from heterogeneous sources, including in vitro studies, animal models, and limited human observational data, which differ substantially in their inferential value. These mechanisms should also be interpreted in light of the exposure route. Inhaled MPs/NPs are primarily deposited in the respiratory tract, where they may induce local inflammation and, depending on their size and properties, translocate into the systemic circulation. In contrast, orally ingested particles mainly interact with the gastrointestinal epithelium and the gut-associated immune system, potentially influencing systemic inflammatory responses through indirect pathways.

Environmental MPs (25–300 µm; 100 ng/mL–1 mg/mL) have been shown to trigger the secretion of pro-inflammatory cytokines, including interleukin-1β (IL-1β) and IL-6, in human monocytic THP-1 cells, a widely used model to investigate monocyte and macrophage function in the cardiovascular system [48,49]. The inflammatory response increased with prolonged exposure and followed a clear dose–response relationship, reaching maximal levels at 1 mg/mL [48].

In THP-1 cells, amine-modified PS particles (50 nm; 50 µg/cm^2^) have also been reported to directly activate the NOD-like receptor protein 3 (NLRP3) inflammasome, a pathway implicated in atherosclerotic inflammation, thereby promoting the release of IL-1β and IL-18 [50,51]. However, other particle types, including PET, polyacrylonitrile, and polyamide 6 (nylon), were able to stimulate IL-8 secretion even in NLRP3 knockout cells, suggesting that MPs may also activate alternative pro-inflammatory pathways depending on their physicochemical properties [50].

Consistent with these findings, stimulation of myocardial microvascular endothelial (MyEnd) cells with particles at the micro-nano boundary (1 µm; 10^7^ particles/mL) resulted in the upregulation of vascular cell adhesion molecule-1 (VCAM-1) and intercellular adhesion molecule-1 (ICAM-1), indicative of endothelial activation, a hallmark of early atherogenesis [52]. In vivo, C57BL/6N mice injected with plastic particles (1 µm; 2.5 mg) exhibited significantly increased IL-1β levels and marked endothelial activation, as reflected by elevated VCAM-1 and ICAM-1 expression in aortic tissue [52]. The upregulation of adhesion molecules such as ICAM-1 and VCAM-1 facilitates the recruitment and transmigration of circulating monocytes into the vascular wall, where they differentiate into macrophages and contribute to foam cell formation, a hallmark of early atherosclerotic plaque development [53,54].

Long-term exposure studies further support these observations. In male Sprague–Dawley rats, PS-MP exposure (5 µm; 0.5 mg/L for 120 days) induced mild vascular alterations, including increased calcification in small cardiac vessels and the ascending aorta, accompanied by elevated plasma levels of IL-1β, IL-6, and tumor necrosis factor alpha (TNF-α) [55]. Similarly, Wistar rats exposed for 90 days to PS-MPs (0.5–50 mg/kg/day) showed increased myocardial enzyme levels and elevated expression of inflammatory markers (IL-1, IL-1β, ICAM-1) in cardiac and aortic tissues, although no overt histopathological changes were observed [56]. Mechanistically, PS-MP exposure activates the caspase-1-dependent NF-κB/NLRP3/gasdermin D (GSDMD) signaling axis, promoting pyroptosis, an inflammatory form of programmed cell death potentially contributing to vascular inflammation and CVD progression [57,58]. This was confirmed by increased levels of cleaved GSDMD, IL-1β, and IL-18 in cardiac tissue in male Wistar rats, alongside evidence of cardiomyocyte pyroptosis [57]. Chronic activation of inflammasome pathways and pyroptotic cell death may further contribute to processes associated with plaque instability by promoting necrotic core formation and weakening the fibrous cap, increasing the risk of plaque rupture and acute cardiovascular events [59,60].

Taken together, these findings suggest that MP/NP-induced inflammation may contribute to key pathophysiological processes relevant to CVD, including endothelial dysfunction, immune activation, and vascular remodeling. From a physiological perspective, these alterations may directly impair endothelial function by reducing NO bioavailability, a key regulator of vascular tone and homeostasis [61]. Decreased NO levels promote vasoconstriction, leukocyte adhesion, and platelet activation, thereby contributing to early atherogenesis and increased thrombotic risk [62]. On the other hand, not all inflammatory responses induced by MPs/NPs necessarily translate into CVD risk. While processes such as sustained endothelial activation, monocyte recruitment, foam cell formation, and vascular remodeling are directly implicated in atherogenesis, transient or isolated increases in inflammatory biomarkers may reflect nonspecific responses to foreign particles. In this context, inflammatory activation should be interpreted with caution unless it is supported by evidence of persistence, dose–response relationships under physiologically relevant conditions, and clear linkage to downstream pathological endpoints.

Human data, although still limited, further support the potential relevance of these findings. In patients with detectable MPs in carotid artery plaques (PE and PVC) and in coronary blood (PS and PVC), MP presence was associated with elevated inflammatory markers, including IL-1β, IL-18, IL-8, and TNF-α, as well as increased plaque collagen content and higher levels of CD3 and CD68, markers of lymphocyte and macrophage infiltration, respectively [38,39]. Furthermore, in patients with ACS) circulating MP levels were positively associated with IL-6 and IL-12p70, as well as with increased B-cell and natural killer cell abundance [40]. Notably, different MP types exerted distinct immunological effects, likely reflecting their specific physicochemical characteristics [40].

MPs may modulate inflammatory responses relevant to tumor-related processes. In skin squamous cell carcinoma models (SCL-1 and A431), exposure to PE-MPs (≤1 mg/mL) promoted cell proliferation in a dose- and time-dependent manner through activation of the NLRP3 inflammasome via a mitochondrial DNA (mtDNA)-mediated mechanism [63]. In contrast, treatment of normal human keratinocytes (HaCaT cells) with PE-MPs inhibited proliferation and induced pyroptosis through the same mtDNA–NLRP3 signaling axis, suggesting that MPs may exert divergent effects by promoting proliferation in tumor growth while inducing damage in healthy cells [63]. Although these findings derive from in vitro models, they may be relevant to dermal exposure scenarios, which represent a potential route of contact for MPs/NPs, although their effective penetration through intact skin remains limited and incompletely characterized.

In lung cancer models, exposure to submicron particles (800 nm; 10–500 µg/mL, 48 h) induced cellular senescence in A549 human lung adenocarcinoma cells, as evidenced by enlarged morphology, irreversible cell cycle arrest, increased SA-β-gal activity, and metabolic dysregulation [64]. This phenotype was accompanied by increased expression of IL-1α, IL-1β, IL-6, and IL-8/CXCL8, key components of SASP [64]. While SASP can reinforce growth arrest via autocrine and paracrine signaling, it may also promote tumorigenic processes, including angiogenesis, stemness, genotoxicity, chronic inflammation, invasion, migration, and immunosuppression, representing a well-recognized “double-edged sword” in cancer biology [65]. Accordingly, particle-induced senescence and associated inflammatory signaling may be associated with impaired tissue repair and persistent cellular alterations in lung tissue [64].

In vivo evidence, although still limited, supports the role of MP/NP-induced inflammation in shaping tumor-promoting microenvironments. In a model of an azoxymethane/dextran sodium sulfate (AOM/DSS)-induced colitis-associated cancer model, BALB/c mice exposed to 20 nm PS-NPs (0.1; 1.0; 20 mg/kg/day for two weeks) exhibited larger adenocarcinomas and greater infiltration of inflammatory cells than those in the normal control group [66]. Oral administration of PS-NPs (10 mg/kg/day for 7 days) in BALB/c mice impaired intestinal barrier function and increased infiltration of IL-1β-producing macrophages in the colon [67]. This was associated with polarization of CD4^+^ T cells toward Th17 and Treg subsets, along with T-cell exhaustion, collectively contributing to the establishment of a pro-tumorigenic microenvironment under experimental conditions [67].

Inflammation emerges as a key biologically plausible mechanism potentially linking MP/NP exposure to disease. Across both cardiovascular and cancer contexts, evidence frequently reports the involvement of NLRP3 activation and cytokine-driven inflammatory signaling as key shared pathways. However, most of the available evidence derives from experimental models, often involving exposure levels exceeding those encountered under real-world conditions, thereby limiting direct extrapolation to human disease. Current human evidence is primarily associative, indicating correlations between MP presence and inflammatory markers, but does not establish causality. Accordingly, while inflammation represents a plausible mechanistic pathway, its relevance to disease onset and progression in humans remains to be clarified. Similarly to CVD, inflammation alone is not sufficient to promote tumor development. The oncogenic relevance of MP/NP-induced inflammation depends on its interaction with additional processes, including oxidative stress, genomic instability, epigenetic alterations, immune dysregulation, and remodeling of the tumor microenvironment. Consequently, inflammatory responses should be interpreted as potentially contributing factors rather than direct evidence of cancer initiation or progression. Therefore, while MP/NP-induced inflammatory responses may contribute to tumor-promoting conditions, they should not be interpreted as evidence of carcinogenic potential in the absence of complementary mechanisms such as genomic instability, sustained proliferative signaling, or evasion of cell death.

Overall, the available evidence is more extensive for cardiovascular outcomes, supported by mechanistic, in vivo, and emerging human data, although important limitations remain, particularly regarding exposure relevance and causality. In contrast, evidence for cancer remains limited due to the lack of longitudinal human studies and the reliance on in vitro and early in vivo models.

### 3.2. Oxidative Stress

Oxidative stress, defined as an imbalance between reactive oxygen species (ROS) and antioxidant defenses generated by both endogenous metabolic processes and exogenous exposures, represents a central and multifaceted mechanism potentially linking MP/NP exposure to cellular dysfunction [10,33,68]; however, its interpretation requires integration within a broader redox biology framework. ROS originate from multiple intracellular sources, including mitochondria, NADPH oxidases, and other enzymatic systems [69]. Additional contributors include uncoupled endothelial nitric oxide synthase (eNOS), xanthine oxidase, and endoplasmic reticulum-associated oxidoreductases, which may further amplify oxidative stress under pathological conditions [69]. Importantly, ROS generation is highly compartmentalized: mitochondrial ROS are mainly associated with metabolic regulation, apoptosis, and bioenergetics, whereas cytosolic ROS—largely derived from NADPH oxidases—are more directly involved in signaling and inflammatory pathways [69]. Antioxidant defenses comprise both enzymatic systems, such as superoxide dismutases (SODs), catalase (CAT), glutathione peroxidases (GPxs), peroxiredoxins, and thioredoxin systems, and non-enzymatic components, including glutathione (GSH), vitamins C and E, and other small-molecule antioxidants [70]. In addition to absolute GSH levels, the GSH/GSSG ratio is widely regarded as a sensitive indicator of intracellular redox status and antioxidant capacity [71]. These systems act in a coordinated manner to detoxify ROS and preserve intracellular redox homeostasis [70]. A central regulator of this adaptive antioxidant response is the nuclear factor erythroid 2-related factor 2 (Nrf2)/Keap1 signaling pathway, which controls the transcription of numerous cytoprotective genes involved in antioxidant defense, detoxification, and cellular resilience to oxidative stress [72,73]. When redox homeostasis is disrupted, excessive ROS production leads to lipid peroxidation, generating reactive aldehydes such as malondialdehyde (MDA) and 4-hydroxynonenal, which reflects oxidative modification of DNA bases, and phosphorylated histone H2AX (γ-H2AX), which serves as a marker of DNA double-strand breaks and genotoxic stress [74,75,76]. In this context, ferroptosis—an iron-dependent form of regulated cell death driven by lipid peroxidation—has emerged as a potential mechanism linking oxidative stress to tissue injury. Importantly, changes in antioxidant systems should be interpreted within a dynamic framework. Upregulation of antioxidant enzymes or Nrf2 activation may reflect adaptive or compensatory responses at low or moderate exposure levels, whereas sustained or excessive exposure may lead to antioxidant exhaustion and irreversible oxidative damage [77]. This dose- and time-dependent transition is consistent with the concept of redox hormesis [77]. Accordingly, increases in antioxidant enzymes such as SOD, CAT, and GPx, as well as activation of Nrf2-dependent pathways, should not be interpreted per se as evidence of toxicity or irreversible cellular damage. Rather, these responses may reflect adaptive mechanisms aimed at restoring redox homeostasis and should be evaluated in conjunction with markers of oxidative injury, antioxidant depletion, cellular dysfunction, exposure duration, and dose.

Within this redox framework, oxidative stress is a major driver of CVD development [10,33]. Given the abundance of mitochondria in cardiomyocytes and their essential role in ROS production, mitochondrial dysregulation predisposes to CVD onset [10]. Excessive ROS generation damages cellular lipids, proteins, and DNA, alters signaling pathways, compromises genomic integrity, and promotes tumor initiation and progression [33]. In addition, oxidative stress-induced reductions in NO bioavailability and increased ROS production contribute to endothelial dysfunction, vascular stiffness, and hypertension [78]. These alterations may also promote myocardial remodeling, fibrosis, and impaired contractile function through redox-sensitive signaling pathways [79].

Oxidative stress has also emerged as one of the most frequently reported adverse effects of MP/NP exposure [68]. Elevated ROS levels promote lipid peroxidation, protein oxidation, mitochondrial dysfunction, and DNA damage, ultimately disrupting cellular redox homeostasis and suggesting a potential link between MP/NP exposure and systemic toxicity through inflammation, cell death, and organ dysfunction [68,80]. The induction of oxidative stress by MPs/NPs may also depend on the route of exposure and subsequent tissue distribution. For example, inhaled particles may directly affect pulmonary and vascular redox balance, whereas orally ingested particles primarily exert effects at the intestinal level, with potential secondary systemic consequences following translocation.

Exposure to NPs (25 nm; 0.1–10 µg/mL for 24 h) induces premature cellular senescence in porcine coronary artery endothelial cells, as shown by upregulation of p53, p16, and p21, increased SA-β-gal activity, and reduced eNOS expression, consistent with enhanced oxidative stress [81]. NP treatment downregulates Sirt1, a key stress and energy sensor activated by an increased NAD^+^/NADH ratio, thereby promoting endothelial dysfunction and NADPH oxidase-dependent ROS production, ultimately contributing to vascular senescence and atherosclerosis development [81,82,83,84]. In human embryonic stem cell-derived cardiomyocytes, PS-NP exposure (20–40 µg/mL for 7 days) increased CAT expression and superoxide anion levels, indicating oxidative stress independent of the SOD pathway [85,86]. ROS accumulation triggered endoplasmic reticulum stress and apoptosis, as evidenced by increased cleaved caspase-3 levels, leading to irregular contractile rhythms [85,87].

In vivo, zebrafish embryos exposed to NPs (25–1000 µg/mL) developed pericardial edema, impaired angiogenesis, and dose-dependent cardiovascular dysfunction, including reduced cardiac output and blood flow velocity, with an increased risk of thrombosis [88]. These alterations were associated with ROS overproduction, endothelial barrier disruption, and a pro-inflammatory, procoagulant state [88]. Temperature further modulated NP toxicity (PS-NH_2_ NPs toxicity: 50 nm; 0.1 mg/L), partially improving cardiac performance while exacerbating oxidative stress and mortality, highlighting the interaction between environmental factors and NP-induced cardiotoxicity [89].

Consistent findings were observed in Wistar rats, where 90-day exposure to PE-MPs (5 and 50 mg/L) increased MDA levels and reduced SOD, CAT, and GPx activities, indicating oxidative imbalance [90]. Such dysregulation may activate the Wnt/β-catenin pathway, a key regulator of cardiac function, promoting profibrotic and hypertrophic remodeling [90,91]. Similarly, long-term intragastric administration of high concentrations of PS MPs (50 mg/kg/day for 90 days) in male Wistar rats induced myocardial damage associated with reduced antioxidant defenses (decreased SOD and GSH levels) and increased lipid peroxidation (elevated MDA levels) [56]. In male adult C57BL/6 mice, chronic exposure to MPs via drinking water (5 µm; 1000 µg/L for 180 days) worsened vascular lesions and cardiac abnormalities, partially attributable to oxidative stress [92].

Additional evidence from aquatic models supports this mechanism. In carp, PS-NP exposure (50–400 nm; 1000 µg/L) increased ROS and MDA levels while impairing antioxidant defenses [93]. These alterations were associated with activation of the TLR4/NOX2 pathway, Th1-skewed immune responses, and apoptosis via the IGFBP3/p53/ACHE axis, leading to size-dependent myocardial injury, with greater toxicity observed for smaller particles [93].

Experimental studies also indicate a role for oxidative stress in MP/NP-associated carcinogenic processes. Exposure to PS submicron particles (0.5 µm; 5 µg/mL, 48 h) or chronic treatment with PS submicron particles (0.5 µm) or PS-MPs (2 µm; 20 µg/mL, 4 weeks) increased ROS generation in normal human intestinal CCD-18Co cells, driving metabolic reprogramming toward enhanced glycolysis, lactate production, and glutamine utilization [94]. Notably, this metabolic shift parallels the changes induced by the carcinogen AOM and observed in HCT15 colon cancer cells, highlighting a canonical cancer-associated adaptation involving the decoupling of glucose and glutamine metabolism under stress conditions [94]. Treatment of lipopolysaccharide (LPS)-stimulated human colorectal adenocarcinoma Caco-2 cells with PS-NPs (20 nm; 125, 250, and 500 µg/mL) for 24 h induced lipid peroxidation and increased intracellular ROS levels [66]. In Caco-2 cells, exposure to PS particles spanning the nano- and microplastic size range (0.1 and 5 µm; 200 mg/mL for 12 h) also significantly increased intracellular ROS generation without detectable cytotoxicity, whereas lower concentrations (≥20 mg/mL for 0.1 µm and ≥1 mg/mL for 5 µm) induced mitochondrial depolarization, with larger particles exerting greater toxicity [95]. In contrast, PE-MP exposure (5–60 µm; 0.25–1.0 mg/mL for 48 h) in Caco-2 and HT-29 cells increased cytotoxicity and oxidative stress primarily through a dose-dependent rise in mitochondrial superoxide production, without affecting total ROS or cytosolic superoxide levels [96]. Ethanol extracts of PE particles further demonstrated that leached chemicals contribute to toxicity by inducing both total ROS and mitochondrial superoxide generation in a cell line-dependent manner [96]. Although these differential responses warrant further investigation, they support the ability of MPs to induce oxidative stress-related toxicity under high-exposure conditions.

In skin squamous cell carcinoma models (SCL-1 and A431), exposure to small PS-MPs (1 µm; 0.25–1 mg/mL) promoted proliferation in a dose- and time-dependent manner via activation of the ROS–mtDNA–NLRP3 signaling pathway [63]. Increased mitochondrial ROS disrupted membrane potential and promoted the release of oxidized mtDNA, as indicated by 8-oxo-2′-deoxyguanosine, which subsequently triggered inflammasome activation and sustained tumor-promoting inflammatory signaling [63].

In human lung adenocarcinoma A549 cells, exposure to PS-NPs (10–25–50–100–250–500 µg/mL) for 24, 48, or 96 h induced a significant, dose- and time-dependent increase in hydrogen peroxide production at all tested concentrations [64]. Concomitantly, increased expression of antioxidant enzymes, including SOD1/2, CAT, GPx1, and heme oxygenase-2, was observed, consistent with activation of oxidative stress-responsive defense pathways [64]. Similarly, exposure of human bronchial epithelial and mesothelial cells (BEAS-2B and CRL-9609) to PS-NPs (80 nm; 100 µg/L for 24 h) increased ROS and MDA levels, accompanied by upregulation of antioxidant genes (*SOD*, *CAT*, *GPX*, and *Nrf2*) and depletion of GSH, indicating activation of the Keap1–Nrf2 adaptive response together with evidence of impaired antioxidant reserve and oxidative injury [97]. PS-NPs also impaired mitochondrial function, as evidenced by membrane depolarization and calcium dysregulation, further amplifying ROS production [97]. Elevated ROS levels promoted inflammation, DNA damage, and necrosis, thereby potentially contributing to lung disease, including cancer [97]. Moreover, integrin α5β1 overexpression enhanced NP internalization and exacerbated oxidative damage, amplifying downstream inflammatory and genotoxic responses and suggesting a potential role for this receptor in mediating NP-induced toxicity [97].

In vivo, administration of PS-NPs (20 nm; 0.1–10 mg/kg) in an AOM/DSS-induced colorectal cancer model in BALB/c mice resulted in a significantly higher number of tumor nodules, accompanied by increased ROS production compared with the control and AOM/DSS groups [66]. In healthy male Sprague–Dawley rats exposed to PS-NPs (varying particle sizes; 5 mg/L/day), gastric tissues exhibited oxidative stress coupled with DNA damage, a marker of potential carcinogenic effects [98]. Notably, SOD, CAT, and GPx activities were reduced, whereas MDA levels were increased, with significant alterations observed exclusively in association with the smallest PS-NPs (80 nm) [98], consistent with previous observations [93].

In summary, oxidative stress emerges as a pivotal mechanism underlying MP/NP-induced toxicity across both cardiovascular and cancer contexts. Evidence from experimental models consistently supports a key mechanistic role for redox imbalance in driving cellular dysfunction. However, most of the available data derive from in vitro and in vivo studies conducted under exposure conditions that may not reflect real-world human scenarios, thereby limiting direct extrapolation. Overall, oxidative stress represents a converging biological pathway linking environmental exposure to downstream pathological processes, highlighting the central role of redox imbalance in plastic-induced toxicity and suggesting its potential as a therapeutic target for mitigation strategies.

Antioxidant interventions, particularly N-acetylcysteine (NAC), have been shown to attenuate MP/NP-induced ROS production, lipid peroxidation, mitochondrial dysfunction, inflammatory signaling, and apoptosis, while restoring endogenous antioxidant defenses, including antioxidant enzyme activity [80,99,100]. In parallel, NAC suppresses the activation of redox-sensitive signaling pathways, such as the pro-inflammatory transcription factor NF-κB and p38 mitogen-activated protein kinase, following exposure to plastic particles [101,102]. This evidence, in addition to suggesting that modulation of redox pathways may mitigate MP/NP-induced toxicity, further supports the role of oxidative stress as a central mechanistic pathway of MP/NP-induced toxicity, rather than a secondary consequence. However, the available evidence remains largely preclinical, and the efficacy of antioxidant strategies in humans has yet to be established. Future studies should further investigate whether modulation of antioxidant pathways may represent a viable strategy to counteract MP/NP-induced biological effects.

### 3.3. Gut Dysbiosis

The human gastrointestinal tract harbors a diverse microbial community (microbiota) comprising approximately 100 trillion microorganisms, representing the largest microbial consortium in the body [103,104]. Disruption of gut microbiota homeostasis (dysbiosis), characterized by alterations in microbial composition and metabolite profiles, is associated with immune dysregulation and chronic inflammation, processes that are involved in the development of various diseases, including CVD and cancer [103,105].

Microbiota-derived metabolites, such as trimethylamine N-oxide and bile acids, have been implicated in processes involved in CVD pathogenesis, including endothelial dysfunction and atherosclerosis [106,107]. In addition, short-chain fatty acids (SCFAs), key metabolites derived from microbial fermentation, and LPS, a potent endotoxin with pro-inflammatory properties, are key microbiota-derived mediators involved in the regulation of immune homeostasis, intestinal barrier integrity, and systemic inflammation, all of which are central processes in CVD pathophysiology [108,109]. Alterations in microbiota composition and reduced diversity have been associated with the development and progression of heart failure [103]. In addition, dysbiosis has been associated with oncogenic processes and tumor progression, including aberrant signal transduction, DNA damage, epigenetic alterations, and immune suppression, while also acting as a key regulator of the tumor microenvironment, with the potential to exert both pro- and anticancer effects [110,111]. Such alterations are closely linked to gut barrier dysfunction, increased intestinal permeability, and bacterial translocation, which may facilitate systemic exposure to pro-inflammatory microbial products [112,113].

Although research on the interaction between MPs/NPs and the gut microbiota is still in its early stages, both in vitro and in vivo studies have documented that plastic particles can modify microbial composition and exert variable effects on microbial diversity and richness [114]. Moreover, MP/NP exposure has been associated with changes in microbiota-derived metabolites, including SCFA and LPS levels and bile acid profiles, as well as with disruption of intestinal epithelial tight junction proteins (e.g., occludin, claudins, and zonula occludens), potentially resulting in increased gut permeability [114,115,116,117,118]. Importantly, these mechanistic insights remain largely derived from experimental models, and confounding factors such as diet, age, antibiotic exposure, and baseline disease status must be considered when extrapolating these findings to human populations. They are primarily relevant to orally ingested MPs/NPs, which directly interact with the intestinal microbiota and epithelial barrier. In contrast, inhalation exposure is less likely to directly influence gut microbial composition, except in cases where particles are cleared via mucociliary transport and subsequently swallowed or redistributed systemically.

To date, a limited number of studies have addressed cardiovascular endpoints potentially associated with MP-induced gut microbiota alterations, whereas only a single study has examined their relevance in the oncological context. Collectively, recent evidence highlights that gut microbiota alterations and related metabolic changes may play a role in mechanisms potentially involved in cardiovascular effects associated with MP exposure. Yan et al. reported that PS-MPs (5 µm; 0.5 mg/L) may induce increased vascular calcification in male Sprague–Dawley rats through perturbation of the gut microbiota, impairment of the intestinal barrier, and inflammatory responses [55]. Exposure resulted in a marked increase in Proteobacteria, a major source of LPS endotoxin, and in the dominance of *Escherichia_Shigella* at the genus level, while non-exposed animals were enriched in SCFA-producing taxa, which contribute to gut microbiota homeostasis and intestinal barrier integrity. These findings are consistent with a mechanistic link between MP-induced gut dysbiosis and vascular calcification [55]. The same study reported higher concentrations of MPs in fecal samples from patients with vascular calcification than in those without, supporting potential clinical relevance of these findings [55]. Consistently, Wang et al. showed that oral exposure to PS-MPs (5 µm; 1–2 mg/L) for 8 weeks in BALB/c mice led to hypertension and cardiac injury, characterized by increased systolic, diastolic, and mean blood pressure, as well as cardiac hypertrophy and fibrosis [119]. These effects might be mediated by microbiota alterations, as evidenced by the enrichment of specific bacterial genera, including *Candidatus Arthromitus*, *Akkermansia*, *Anaeroplasma*, and *Prevotella*, which positively correlated with cardiovascular injury markers [119]. Of note, modulation of the gut microbiota through a high-fiber diet or antibiotic treatment significantly attenuated these alterations, while fecal microbiota transplantation from MP-exposed donors transferred the hypertensive phenotype and exacerbated cardiovascular damage in recipient mice, supporting a potential mechanistic role of gut dysbiosis in experimental models [119]. The authors also reported higher concentrations of PS-MPs (up to 30 µm) in hypertensive patients compared with normotensive individuals, further supporting the relevance of gut microbiota alterations and associated metabolic changes as potential mechanistic mediators relevant to the association between MP exposure and CVD-related processes [119]. In line with these findings, Song et al. observed that prolonged exposure to PS-MPs (0.5, 5, and 50 mg/kg/day) in SPF male Wistar rats induced dose-dependent shifts in systemic metabolic profiles, with significant upregulation of metabolites with antioxidant and anti-inflammatory properties, including equol and 4-hydroxybenzoic acid, suggesting adaptive microbiota-related metabolic responses [56].

In the oncological framework, Tian et al. further confirmed that exposure to PS-NPs may induce significant alterations in gut microbial composition, potentially relevant to cancer-related processes [66]. In an AOM/DSS mouse model, PS-NPs (20 nm; 10 mg/kg) caused significant changes in gut microbiota diversity, with enrichment of *Allobaculum*, a colitogenic bacterium, and a decreased abundance of *Lactobacillus*, thereby weakening its cancer-protective effects [66]. Both *Allobaculum* and *Lactobacillus* species are involved in lipid metabolism, suggesting that PS-NPs may partly affect metabolic processes through microbiota modulation in this model [66].

Overall, current evidence suggests that MP-induced gut microbiota dysbiosis and related metabolic alterations may represent a relevant mechanistic pathway linking MP exposure to both cardiovascular and oncological outcomes. However, consistent with other mechanisms discussed, most of the available evidence derives from experimental studies, with only limited human data, and is often affected by substantial heterogeneity in exposure assessment and microbiota characterization. Moreover, the complexity of host–microbiota interactions and the influence of confounding factors—such as diet, antibiotics, and environmental exposures—make it challenging to establish direct causal relationships.

Taken together, microbiota-mediated effects may represent an indirect but potentially important pathway through which MPs/NPs contribute to systemic disease processes, although further studies are required to clarify their causal and translational relevance.

### 3.4. Genotoxicity

DNA damage and defective repair mechanisms are widely recognized as central processes in disease development, including cancer and CVD. Damage to DNA, whether caused by endogenous processes or by external agents, can lead to mutations and genomic instability [120]. In cancer, the accumulation of such genetic alterations in key regulatory genes drives uncontrolled cell proliferation and malignant transformation [121]. Similarly, in CVD, DNA damage in vascular endothelial cells and smooth muscle cells contributes to cellular dysfunction, inflammation, and impaired tissue repair. Over time, these processes promote atherosclerosis, vascular stiffening, and plaque instability [122]. Therefore, genotoxic stress and defective DNA repair mechanisms represent a shared biological foundation underlying both cancer and CVD, linking genomic integrity to long-term disease risk.

The potential genotoxicity of MPs/NPs is thought to arise from their interactions with cellular structures, particularly DNA, potentially leading to genetic damage and impaired cellular function. Such processes are likely influenced by the route of exposure, which determines particle uptake, systemic distribution, and access to intracellular compartments. In particular, orally ingested and inhaled particles may differ in their ability to translocate across biological barriers and reach target tissues where genotoxic effects may occur. These processes are largely attributed to ROS overproduction and direct intracellular interactions, which may compromise chromosomal integrity and disrupt mitotic processes, ultimately contributing to genomic instability [68,123]. Notably, direct evidence of MP/NP-induced genotoxic effects in human tissues remains extremely limited, and most current knowledge is derived from experimental systems.

Consistent experimental evidence shows that MP/NP exposure has been shown to induce DNA strand breaks, chromosomal alterations, and oxidative lesions [16,27]. However, it is important to distinguish between transient or repairable DNA damage and fixed mutations contributing to long-term genomic instability, as this distinction remains insufficiently explored in current studies. Most available studies report markers of DNA damage, oxidative stress, or chromosomal injury, whereas evidence demonstrating mutation fixation, persistence across cell divisions, or alteration of functionally relevant genes remains scarce. Consequently, the mechanistic link between MP/NP-induced DNA damage and cancer development, although biologically plausible, is not yet fully established.

Persistent oxidative stress may overwhelm antioxidant defenses and DNA repair mechanisms, further worsening genomic instability and activating stress-related signaling pathways [123]. Although direct evidence in human cardiovascular systems is absent, these mechanisms are closely associated with vascular aging and atherogenesis.

Increasing evidence suggests a potential link between MP exposure and carcinogenic processes, largely through DNA damage-related mechanisms. Recent findings highlight a mechanistic connection between MPs/NPs, particularly PS-NPs, and the progression of colitis-associated cancer. Tian et al. reported that PS-NPs exacerbated tumor development in association with oxidative stress, metabolic disruption, and DNA damage [66]. In an AOMe/DSS-induced murine model, chronic 20 nm PS-NP exposure, administered by oral gavage at low (0.1 mg/kg), medium (1.0 mg/kg), or high (10 mg/kg) doses, was associated with increased tumor burden, induced DNA damage, and promoted histopathological features consistent with more aggressive tumor phenotypes [66]. Mechanistically, PS-NPs were reported to activate the PI3K/AKT/mTOR signaling pathway, a central regulator of cellular metabolism, proliferation, and survival, in LPS-treated Caco-2 cells [66].

Moreover, in an in vitro study, MEF Ogg1−/− mouse embryonic fibroblasts (deficient in oxidative DNA base repair) were exposed to chronic low-dose PS-NP exposure (50 nm; 25 µg/mL) [124]. After 12 weeks of exposure, the cells exhibited increased DNA damage, enhanced anchorage-independent growth, increased migration and invasion, as well as acquisition of features consistent with malignant transformation features [124].

Ding and colleagues reported that long-term exposure to PS-MPs (5 mg/L for 90 days) in rats resulted in widespread tissue distribution, together with a significant increase in ROS levels, reduced activity of antioxidant enzymes (SOD, CAT, GPx), and increased oxidative DNA damage (8-oxo-dG and phosphorylated histone H2AX) [98].

Collectively, current evidence suggests that MPs/NPs may induce oxidative DNA damage, DNA strand breaks, chromosomal abnormalities, and impaired DNA repair through ROS-mediated mechanisms and intracellular interactions. Nevertheless, evidence demonstrating the persistence of these lesions as fixed mutations and their direct involvement in disease-relevant genetic alterations remains limited.

While these mechanisms are well supported in cancer models, the relevance of genotoxic pathways in CVD remains largely indirect and has not yet been specifically addressed in dedicated experimental or human studies. Moreover, most of the available evidence derives from in vitro systems or high-dose animal exposures, which may not accurately reflect real-world human exposure scenarios, highlighting the need for further research to clarify the relevance of genotoxic pathways in vascular pathology.

### 3.5. Epigenetic Modifications

Epigenetics refers to reversible changes in gene expression mediated by mechanisms such as DNA methylation, histone modifications, and non-coding RNAs, which occur without alterations to the underlying DNA sequence and allow environmental and cellular cues to influence biological function. These changes can also be mitotically heritable, raising important considerations within the framework of the developmental origins of health and disease. Accordingly, epigenetic mechanisms have been proposed as potential mediators through which environmental exposures, including MPs/NPs, may influence biological responses. However, whether MP/NP-induced epigenetic alterations contribute to the development of CVD or cancer in humans remains unclear [16,17].

DNA methylation, mediated by DNA methyltransferases (DNMTs), is essential for normal cellular processes and enables the transmission of regulatory information across cell divisions. Aberrant DNA methylation is recognized as one of the bona fide hallmarks of cancer [125] and can dysregulate pathways involved in coronary heart disease, heart failure, and hypertension, thereby contributing to disease onset and progression [126]. Histone modifications modulate chromatin structure by regulating histone–DNA interactions, influencing gene accessibility and transcriptional activity. Dysregulated histone modification patterns can lead to aberrant gene expression programs, including activation of oncogenes or repression of tumor suppressor genes, processes involved in uncontrolled cell proliferation and malignant transformation [127]. In CVD, abnormal histone post-translational modifications, such as methylation, acetylation, crotonylation, and lactylation, play an essential role in disease pathogenesis by reshaping transcriptional programs without altering the DNA sequence [128]. In addition to these mechanisms, dysregulation of noncoding RNAs, including micro (mi) RNAs, is strongly associated with cancer development and CVD. Pro-inflammatory miRNAs promote atherosclerosis, whereas others exert protective effects in MI [129]. Dysregulation of specific miRNAs further contributes to disease progression, including plaque formation, cardiac fibrosis, and disturbances in cardiac conduction [130,131,132].

Within this context, MPs/NPs have emerged as potential environmental stressors that may induce toxicity, partly through epigenetic changes. By affecting multiple regulatory layers, these particles have been shown to reshape cellular identity and function, potentially contributing to disease pathogenesis. Several studies have reported changes in both global and gene-specific methylation patterns following PS-NP exposure. Consistent with these observations, dysregulation of key enzymes involved in DNA methylation, including DNMT3A, has been reported in human fibroblasts and induced pluripotent stem cells exposed to PS-NPs (50 nm; 50 µg/mL), with potential downstream effects on gene regulatory programs [133]. These changes were associated with alterations in fundamental biological pathways governing pluripotency, oncogenic transformation, and inflammatory signaling, extending beyond nuclear DNA to mtDNA methylation and impairing mitochondrial energy metabolism and redox balance [133]. Despite such evidence, direct links between epigenetic alterations induced by MPs/NPs and disease initiation or progression remain limited. The occurrence of epigenetic changes may also depend on particle biodistribution and cellular uptake, which are influenced by the route of exposure and the physicochemical properties of MPs/NPs. At present, the relationship between specific exposure pathways and epigenetic responses remains largely unexplored.

Zhang et al. reported that N6-methyladenosine (m6A) modification of non-coding RNAs was associated with MP-induced cardiac alterations [134]. The authors observed multi-organ accumulation of MPs, accompanied by increased apoptosis in cardiac cells [134]. In the myocardium, treatment of C57BL/6 male mice with MPs (10 µm; 1000 g/L in drinking water for 180 days) was associated with elevated global m6A levels and upregulation of METTL3 expression. RNA-sequencing further revealed extensive transcriptomic changes in the hearts of MP-exposed mice, with more than 300 long non coding (lnc) RNAs and circular (circ) RNAs differentially expressed [134]. These changes were primarily associated with pathways related to endocytosis, cellular senescence, and cell cycle regulation, suggesting molecular pathways that may be involved in the cardiac response to MP exposure [134]. RNA sequencing analysis of vascular tissues also revealed the differential expression of 674 mRNAs, 39 lncRNAs, 196 miRNAs, and 565 circRNAs in chronically exposed MP-treated mice (~5 µm; 1000 μg/L through their drinking water for 180 days) compared with controls [92]. Consistent with these transcriptomic changes, pathway enrichment analyses highlighted mechanisms associated with MP toxicity, including oxidative stress, apoptosis, and fibrosis [92]. In parallel, exposure to plastics such as PET has been shown to alter the miRNA cargo of serum-derived extracellular vesicles (EVs), with predicted target genes enriched in cardiovascular and metabolic disease pathways, suggesting that MPs/NPs may exert effects beyond directly exposed tissues by propagating through EV-mediated intercellular communication and potentially amplifying systemic toxicity [135]. Specifically, immature pigs were orally administered PET-MPs for 4 weeks at low (0.1 g/day) and high (1 g/day) doses. PET-MP exposure induced dose-dependent changes in the metabolomic profile of serum-derived EVs, with 24 and 31 metabolites significantly altered in the low- and high-dose groups, respectively. These metabolic changes were enriched in pathways involved in lipid signaling, mitochondrial dysfunction, glucose metabolism, and steroidogenesis. Although the evidence remains preliminary, the convergence of epigenetic [135] reprogramming with established cardiovascular pathogenic pathways supports further investigation into MP/NP-induced epigenetic alterations as potential mechanisms contributing to cardiovascular toxicity [136].

Regarding the potential role of epigenetic mechanisms in MP/NP-associated carcinogenic processes, growing experimental evidence indicates that exposure to MPs/NPs can induce epigenetic alterations, including modifications in gene expression and DNA methylation patterns, which may affect pathways relevant to carcinogenesis; however, their direct contribution to cancer development remains to be established [137].

Early life-stage PS-MP exposure (9.5–11.5 μm; 50 mg/L) in a zebrafish model may induce global DNA hypomethylation, a molecular alteration commonly observed in carcinogenesis [138]. Furthermore, PS-MP exposure has been reported to modify miRNA expression profiles, suggesting potential disruption of regulatory pathways involved in the control of oncogenes and tumor suppressor genes [138]. However, the functional relevance of these changes for cancer development remains unclear [138]. Disruption of a cluster of miRNAs associated with oncogenic processes was also observed by Barguilla et al. following long-term (180-day) in vitro exposure of mouse embryonic fibroblasts to 50–100 nm PS-NPs [139]. Most of the affected miRNAs were upregulated, including miR-21, miR-23a, miR-25, miR-30c, miR-30d, miR-96, miR-135b, miR-148b, miR-155, miR-199b, miR-200a, miR-210, miR-218, and miR-502, whereas only miR-34a and miR-203a were significantly downregulated [139].

Taken together, experimental studies indicate that MP/NP exposure can be associated with changes in DNA methylation, RNA modifications, non-coding RNA expression, and EV cargo. These findings provide mechanistic hypotheses regarding how MP/NPs may influence cellular responses to environmental stress. However, most evidence derives from in vitro systems and animal models, and only a limited number of studies have investigated the functional consequences of these molecular alterations. Importantly, many reported epigenetic changes may represent adaptive responses to exposure rather than pathogenic events directly involved in disease development. Currently, there is insufficient evidence to determine whether MP/NP-induced epigenetic alterations contribute to CVD or cancer in humans, and no firm conclusions can be drawn regarding long-term or transgenerational effects. Further studies integrating mechanistic and longitudinal epidemiological data are needed to clarify their biological and clinical relevance.

To facilitate a comparative overview of the evidence supporting the different shared mechanisms identified in the literature, we developed an exploratory semi-quantitative Evidence Mapping Score (EMS), a descriptive tool based on both the type and quantity of available evidence. The score was calculated according to the formula EMS = T × Q, where T represents the type of evidence and Q represents the quantity of evidence.

The type of evidence (T) was categorized according to the experimental model used in the included studies. Cellular studies were assigned a score of 1, animal studies a score of 2, and human studies a score of 3. Evidence derived from multiple experimental models received progressively higher scores, reflecting the convergence of findings across different levels of biological investigation: cellular + animal = 4, cellular + human = 5, animal + human = 6, and cellular + animal + human = 8.

The quantity of evidence (Q) was determined according to the number of available studies addressing a given mechanism. One study was assigned a score of 1, two to four studies a score of 2, and five or more studies a score of 3.

The resulting score was used to classify the relative extent of available evidence into five categories: very limited (1–3), limited (4–6), moderate (7–12), substantial (13–18), and extensive (>18).

This scoring approach was developed as a descriptive tool to summarize and compare heterogeneous mechanistic evidence across different biological domains. It is not intended to represent a formal evidence-grading framework and should not be interpreted as equivalent to established methodologies. The score does not incorporate methodological quality, risk of bias, study size, or other factors typically considered in formal evidence assessment. Furthermore, the weighting assigned to different evidence types was based on expert judgment and has not been formally validated.

Accordingly, the score should be regarded as a descriptive and hypothesis-generating metric designed to support evidence mapping rather than to establish the strength or certainty of evidence. Conclusions regarding biological outcomes were therefore based on the overall interpretation of the literature rather than on the numerical score alone. Nevertheless, the approach provides a transparent and reproducible framework for summarizing a highly heterogeneous body of preclinical and clinical evidence and for identifying areas where evidence convergence is greater or where further research is needed.

Table 1 summarizes the results obtained for each shared mechanism discussed, where the circles represent the corresponding EMS achieved (from 1 to 5 circles).

## 4. Exposure Relevance and Methodological Considerations

The interpretation of experimental findings on MP/NP toxicity requires careful consideration of exposure relevance and methodological constraints.

Despite the growing number of studies reporting the presence of MPs/NPs in human cardiovascular and tumor tissues, these findings should be interpreted with caution due to several methodological limitations. The analysis of MPs/NPs in biological samples is highly susceptible to contamination throughout the analytical workflow, including sample collection, processing, and laboratory handling [140]. Airborne microfibers represent a major source of background contamination, while plastic laboratory consumables, reagents, and infrastructure can introduce additional particles that compromise data reliability and reproducibility [140,141]. Consequently, the implementation of procedural blanks, field blanks, and contamination-control measures is essential to ensure data quality [142]. However, the use and reporting of these controls remain heterogeneous across studies [142]. In addition, the analytical techniques employed differ substantially in their ability to identify plastic particles. While some studies rely primarily on morphological characterization, more robust approaches combine microscopic observation with spectroscopic confirmation, such as Fourier-transform infrared spectroscopy, Raman spectroscopy, or pyrolysis–gas chromatography–mass spectrometry, allowing chemical identification of polymers and reducing the risk of particle misclassification [143,144].

Another important source of variability is represented by detection limits and particle-size thresholds. Most currently available methods reliably detect particles in the micrometer range but have limited sensitivity for NPs, which may result in significant underestimation of the total particle burden [144]. Furthermore, studies report MP abundance using different metrics, including particle counts, particle size distributions, mass concentrations, or polymer-specific measurements, making direct comparisons challenging [143]. The biological interpretation of detected particles also remains uncertain. The presence of MPs in tissues may reflect chronic environmental exposure and progressive bioaccumulation [8]. However, given the widespread use of plastic-containing materials in clinical settings and the persistent methodological challenges associated with MP detection, perioperative contamination, exposure to medical devices, and analytical artifacts cannot be completely excluded and should be carefully considered when interpreting tissue findings [8,145]. Finally, disease-associated alterations in tissue architecture, vascular permeability, inflammatory cell infiltration, extracellular matrix composition, or impaired clearance mechanisms may favor particle retention within pathological tissues [146]. Consequently, the higher MP concentrations reported in atherosclerotic plaques, thrombi, and tumor tissues compared with control tissues do not necessarily demonstrate a causal role in disease pathogenesis and may partially reflect differential particle trapping within diseased microenvironments.

While the existing evidence provides important mechanistic insights, its interpretation is limited by methodological heterogeneity, the lack of standardized dose metrics, and a predominant reliance on in vitro and in vivo models [147]. Many experimental studies employ MP/NP concentrations that exceed currently estimated human environmental exposure levels, often by several orders of magnitude. Although such high-dose models are valuable for identifying potential hazard mechanisms, they may not accurately reflect real-world chronic low-dose exposure conditions [148]. Furthermore, the lack of standardized approaches for estimating internal human MP/NP burdens makes direct quantitative comparisons between experimental doses and human exposures particularly challenging. It should also be noted that many experimental studies rely on exposure methods such as direct gavage or injection, which may fail to accurately capture real-world human exposure pathways (e.g., ingestion, inhalation, or dermal contact). This limitation further complicates the interpretation of organ-specific effects and their relevance to different exposure routes.

Most experimental studies are based on acute or short-term exposure paradigms, whereas human exposure is expected to occur predominantly at low doses over prolonged periods [147]. Differences in exposure kinetics may substantially influence biological responses and limit the direct translational extrapolation of experimental findings to human health outcomes [147]. Furthermore, systemic effects described in experimental models may depend on the ability of MPs/NPs to translocate from primary sites of exposure, such as the lung or gastrointestinal tract, into the circulation. However, the extent and efficiency of such translocation in humans remain incompletely characterized and may vary depending on particle size, physicochemical properties, and exposure conditions.

Additional uncertainty arises from the frequent use of uniform, spherical polystyrene particles in laboratory studies [149,150]. These particles differ considerably from environmentally weathered MPs and NPs, which display substantial heterogeneity in size, shape, polymer composition, surface properties, and pollutant-binding capacity [151]. Weathering processes can further modify particle physicochemical characteristics and biological interactions [152].

NPs may exhibit higher cellular uptake and systemic distribution compared with larger MPs, further complicating direct comparisons across studies that do not clearly distinguish between these particle classes [153,154]. Moreover, the toxicological effects observed in experimental models may not be attributable solely to the polymeric core of MPs/NPs. Plastic additives, adsorbed environmental pollutants, heavy metals, and biofilm formation may all contribute to the observed biological responses, potentially acting in a synergistic manner [155]. When ingested, plastic particles may expose organisms to higher concentrations of these co-occurring contaminants or enhance their bioavailability and toxicity [155].

Finally, the absence of harmonized protocols for MP/NP detection, quantification, and characterization, together with the risk of contamination during sample collection and processing, continues to hamper inter-study comparability and exposure assessment [156,157]. Collectively, these limitations highlight the need for standardized methodologies and caution when extrapolating experimental findings to real-world human exposures and disease risk. At the same time, a more integrated framework linking exposure route, toxicokinetics, and tissue-specific biological responses is essential to improve the physiological relevance and interpretation of current evidence.

## 5. Conclusions and Future Challenges

The evidence summarized in this review supports the emerging concept that MPs/NPs may represent previously underestimated contributors to biological processes relevant to the pathogenesis of both CVD and cancer, which together account for a substantial proportion of global morbidity and mortality. This is of particular importance, since CVD alone is responsible for approximately 32% of all global deaths, while cancer accounts for about 16%, meaning that together they contribute to nearly half of worldwide mortality [31,32].

These particles have been shown, particularly in experimental models, to activate several interconnected mechanisms, including chronic inflammation, oxidative stress, gut microbiota alterations, and genotoxic and epigenetic changes, that are already recognized as central drivers of both carcinogenesis and cardiovascular injury. Among these, oxidative stress emerges as a key and converging mechanism, potentially representing one of the earliest and most consistently observed biological responses to MP/NP exposure and a central hub linking environmental exposure to downstream pathological effects.

Inflammation plays a prominent role in mediating the toxic effects of MPs/NPs, particularly in the cardiovascular system, where human evidence supports an association between MP accumulation and inflammatory biomarkers. In contrast, evidence linking MP/NP-induced inflammation to cancer remains largely based on experimental studies, with limited direct translation to human disease. Similarly, increasing evidence suggests that alterations in gut microbiota composition may contribute to systemic inflammation and cardiometabolic dysfunction, although the role of microbiota-mediated pathways in cancer remains less clearly defined.

Emerging mechanistic insights also indicate that MPs/NPs may contribute to biological processes linked to cancer and CVD through genotoxic and epigenetic mechanisms, including DNA damage, altered DNA methylation patterns, and miRNA dysregulation. However, current data remain largely experimental and require further validation in human populations. In this context, an additional and still underexplored area of interest is clonal hematopoiesis of indeterminate potential (CHIP), a condition characterized by the accumulation of somatic mutations in hematopoietic stem cells and associated with both hematological malignancies and increased cardiovascular risk, partly through enhanced inflammatory signaling [11]. Given that cancer and CVD are key outcomes potentially associated with MP/NP exposure, it is plausible that chronic exposure may influence the emergence or expansion of CHIP clones. However, this possibility remains purely hypothetical, with no direct experimental or epidemiological evidence currently available linking MP/NP exposure to CHIP development or clonal expansion. CHIP should be considered as a future hypothesis that warrants investigation in appropriately designed studies, rather than as a mechanism currently supported by evidence.

Despite the growing body of mechanistic evidence, important uncertainties remain, particularly regarding exposure assessment and standardization. Addressing these methodological limitations will be essential for improving exposure characterization and strengthening causal inference in future studies [158].

Further research should prioritize large-scale epidemiological studies integrating accurate exposure assessment, validated biomarkers, and long-term clinical outcomes. In this context, further investigation into antioxidant defense systems and redox-regulating pathways is warranted, particularly to determine whether antioxidant-based strategies may mitigate MP/NP-induced biological damage and reduce disease risk.

Finally, although definitive causal evidence is still lacking, current data suggest that MPs/NPs should no longer be considered biologically inert environmental contaminants. Rather, MPs/NPs may represent emerging components of the exposome with biological plausibility to influence complex disease-related pathways shared by CVD and cancer. However, the magnitude of their contribution to human disease risk and their clinical relevance remain uncertain.

Importantly, the evidence reviewed in this manuscript derives from heterogeneous experimental systems, including in vitro cellular models, animal studies, and human investigations. These models provide complementary insights into molecular mechanisms and biological plausibility; however, their ability to support clinical inference differs substantially. While cellular and animal studies are particularly valuable for identifying mechanistic pathways and establishing biological plausibility, evidence directly linking MP/NP exposure to clinical outcomes in humans remains limited and requires confirmation through well-designed epidemiological and longitudinal studies.

These findings should therefore be interpreted with caution, as causal relationships in humans have not yet been established. Such evidence highlights the need for coordinated public health and regulatory strategies targeting plastic production, waste management, food packaging, and environmental contamination. Bridging current knowledge gaps through interdisciplinary research will be critical to define the true impact of MPs/NPs on human health and to support evidence-based preventive strategies.

## Figures and Tables

**Table 1:** Qualitative assessment of the evidence linking micro- and nanoparticle exposure to cardiovascular and oncological outcomes.

References	Cancer Evidence	References	Cardiovascular Evidence	Mechanism
[63,64,67]	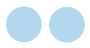 (in vitro + limited in vivo)	[38,39,40,48,50,55,56,57]	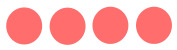 (in vitro + in vivo + prospective human)	Inflammation
[63,64,66,94,95,96,97,98]	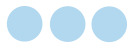 (mechanistic and functional evidence in vitro and-model in vivo)	[56,81,85,88,89,90,92,93]	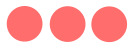 (mechanistic and functional evidence in vitro and-model in vivo)	Oxidative stress
[66]	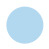 (limited in vivo)	[55,56,119]	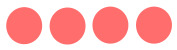 (in vivo + humans)	Gut dysbiosis
[66,98,124]	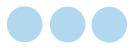 (in vitro + in vivo)	[123]	NA (only indirect experimental evidence)	Genotoxicity
[138,139]	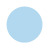 (limited in vitro and in vivo)	[92,133,134,135]	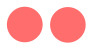 (in vitro + limited in vivo)	Epigenetic modifications

Abbreviations: one circle = very low; two circles = low; three circles = moderate; four circles = high; five circles = very high; NA: not available.

## Data Availability

No new data were created.

## Abbreviations

The following abbreviations are used in this manuscript:

ACS	Acute coronary syndrome
BPA	Bisphenol A
CAT	Catalase
CVD	Cardiovascular disease
CHIP	clonal hematopoiesis of indeterminate potential
DNMT	DNA methyltransferase
GPx	Glutathione peroxidase
IL	Interleukin
LPS	Lipopolysaccharide
MDA	Malondialdehyde
MI	Myocardial infarction
MP	Microplastic
NP	Nanoplastic
NO	Nitric oxide
PA66	Polyamide 66
PE	Polyethylene
PET	Polyethylene terephthalate
PP	Polypropylene
PS	Polystyrene
PVC	Polyvinyl chlorine
ROS	Reactive oxygen species
SA-β-gal	Senescence-associated β-galactosidase
SASP	Senescence-associated secretory phenotype
SOD	Superoxide dismutase
TNF-α	Tumor necrosis factor alpha
